# Isolated Paralysis of the Adductor Pollicis: A Case Report

**DOI:** 10.4061/2011/321020

**Published:** 2011-01-27

**Authors:** F. De Maio, S. Bisicchia, P. Farsetti, E. Ippolito

**Affiliations:** Department of Orthopaedic Surgery, University of Rome “Tor Vergata”, Viale Oxford 81, 00133 Rome, Italy

## Abstract

We report a case of isolated paralysis of the right adductor pollicis in a 30-year-old woman. Electromyographic study showed involvement of the deep motor branch of the ulnar nerve. A ganglion and an anomalous muscle were both ruled out clinically and by MRI as a possible cause of the paralysis. At surgical exploration, we found a fibrous band joining the pisiform and the hook of the hamate bone that compressed the deep motor branch of the ulnar nerve. The fibrous band was excised, and a neurolysis of the motor branch of the ulnar nerve was performed. At followup, eight months later, the patient had fully recovered strength of the adductor muscle.

## 1. Introduction

Both sensory and motor dysfunction as well as a combined dysfunction of the ulnar nerve may be caused by compression of the nerve at Guyon's canal. Ganglion is the most frequent cause of combined dysfunction and of chronic isolated motor deficit [1]. Other causative factors, like anomalous muscle, occupational pressure neuritis, and variation of the path of the deep motor branch of the ulnar nerve at the wrist, which occur less frequently, have also been described [2–5].

We reported a patient who had an isolated paralysis of the right adductor pollicis, caused by a compression to the deep motor palmar branch of the ulnar nerve from a thick fibrous band bridging the pisiform to the hook of the hamate bone. Resection of the fibrous band as well as neurolysis of the motor palmar branch of the ulnar nerve allowed full recovery of muscle function in about eight months.

## 2. Case Report

A 30-year-old woman, whose right side was dominant and who worked as a computer operator, presented with a four-month difficulty in performing some activities of daily life with her right hand like writing, holding suspended objects, and turning a key in a lock. In addition she had noticed dorsal prominence of the first metacarpophalangeal joint. No trauma was referred in her recent history.

At physical examination, the patient showed moderate atrophy of the thenar eminence and of the first web space, an increased dorsal prominence of the first metacarpophalangeal joint, and a positive Froment's sign. Weber's test for the tactile discrimination of two points was negative, as was also Allen's test to evaluate vascularization of the hand. The function of the interossei and hypothenar muscles was normal. 

An electromyography of the muscles of the thenar and hypothenar eminence showed selective dysfunction of the right adductor pollicis (Table 1), but a comparative CT-MRI of the two hands did not reveal any evident causative factor.

**Figure 1:**
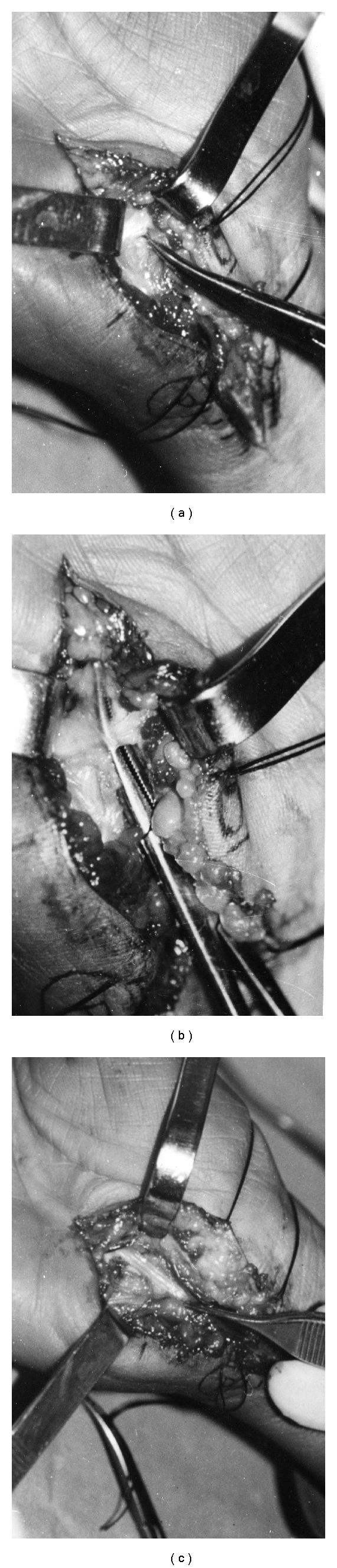
A thick fibrous band crossing over the motor branch of the ulnar nerve was identified at the middle portion of Guyon's canal (indicated by the haemostat) (a) and (b). After the resection of the fibrous band, the deep motor branch of the ulnar nerve appeared decompressed (c).

A surgical exploration of the ulnar nerve at Guyon's canal was proposed to the patients. The operation was performed using a pneumatic tourniquet placed at the arm about 10 cm above the elbow inflated to 300 mmHg. A peripheral block anesthesia of both the medial and ulnar nerve was performed. At surgery, the division of the ulnar nerve was like the pattern A described by Lindsey and Watumull [6], in which the bifurcation into a main sensory trunk and a motor branch occurred proximal to the Guyon's canal. We found only the deep motor palmar branch of the ulnar nerve compressed by a fibrous band joining the pisiform to the hook of the hamate bone in proximity of its emergence from the main trunk. This fibrous band was isolated and excised, and neurolysis of the nerve was also performed (Figure 1).

**Figure 2:**
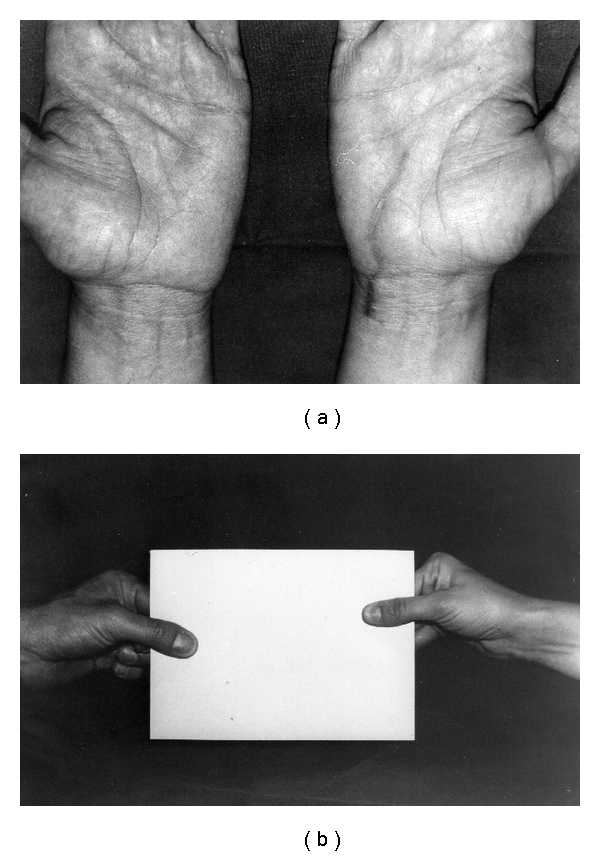
The surgical scar eight months after surgery (a), when Froment's sign became negative (b).

Active movements of the right thumb were started as soon as the patients had recovered from the nerve block anaesthesia, whereas active exercises to improve the strength of the intrinsic muscles of the hand were started the tenth day after surgery. At the final followup, eight months later, the patient had recovered full strength in the adductor muscle, Froment's sign was negative, and the prominence of the first metacarpophalangeal joint had disappeared (Figure 2).

A repeat EMG and nerve conduction velocity was done and demonstrated full recovery of the adductor pollicis (latency: from 3.4 msec pre-op. to 3.0 msec post-op.; amplitude: from 1.6 mV pre-op. to 6.4 mV post-op.)

## 3. Discussion

Compression of the motor branch of the ulnar nerve usually causes weakness and atrophy of the hypothenar muscles and also other muscles such as the interossei, third and fourth lumbricals, adductor pollicis, and part of the flexor pollicis brevis, whereas no sensory impairment is present [7].

A ganglion, accessory muscles, fracture of the hook of the hamate bone, or hypertrophic scars caused by repeated microtrauma are the most common causes of compression of the deep motor branch of the ulnar nerve [8–10].

In our case, the deep motor branch of the ulnar nerve was compressed by a thick fibrous band that joined the hook of the hamate bone to the pisiform.

Some authors have identified this fibrous band as an extension of the tendinous arch, where flexor, abductor, and opponens of the little finger take part of their origin, and have described a compression of the deep motor palmar branch at this level by ganglia and anomalous muscles. This condition spared sensory involvement but caused weakness of all muscles supplied by this nerve, sometimes with the exception of the hypothenar muscles [11].

We believe that in our case the location and the thickness of the fibrous band were abnormal and that the band compressed the nerve in the absence of any other anatomical abnormality. The fibrous band was also screeching on the cut surface. As far as we know, a similar cause of compression has rarely been reported [11, 12].

The clinical peculiarity of our case consisted in the isolated paralysis of the right adductor pollicis muscle, whereas the other muscles innervated by the deep motor branch of the ulnar nerve were spared. We believe that the moderate atrophy of the thenar muscles, observed clinically, could be related to a dysfunction of the deep head of the flexor pollicis brevis, which is innervated by the deep motor branch of the ulnar nerve. Flexion of the thumb was not impaired because the superficial head of the flexor pollicis brevis and the flexor pollicis longus are innervated by the median nerve. The patient's clinical history did nor clarify the cause of the thickening of the tendinous arch of the flexor brevis digiti quinti muscle. However, the patient had completely recovered from the paralysis of the adductor pollicis at the last followup eight months after surgery; furthermore, she showed full electromyographic recovery and had regained complete functionality of the hand.

## Figures and Tables

**Table 1:** Preoperative EMG/NCS of the patient showed selective dysfunction of the right adductor pollicis.

	Latency (msec)	Velocity (m/sec)	Amplitude
ULNAR RIGHT (SAP)	1.8	54.2	8.4 *μ*V
ULNAR LEFT (SAP)	1.9	53.2	9.3 *μ*V
ULNAR RIGHT (cMAP)			
Abductor digiti minimi	2.7	55.2	7.8 mV
Adductor pollicis	**3.4**		**1.6** **mV**
ULNAR LEFT (cMAP)			
Abductor digiti minimi	2.6	58.2	7.3 mV
Adductor pollicis	3.1		6.2 mV
